# 2,6-Bis(2-chloro­ethyl)-8b,8c-diphenyl­perhydro-2,3a,4a,6,7a,8a-hexa­azacyclo­penta­[*def*]fluorene-4,8-dithione

**DOI:** 10.1107/S1600536809024118

**Published:** 2009-06-27

**Authors:** Yandong Wu, Yichong Sun

**Affiliations:** a Key Laboratory of Pesticides and Chemical Biology of the Ministry of Education, College of Chemistry, Central China Normal University, Wuhan 430079, People’s Republic of China

## Abstract

In the title mol­ecule, C_24_H_26_Cl_2_N_6_S_2_, the two phenyl rings form a dihedral angle of 51.95 (7)° and the distance between their centroids is 4.156 (8) Å. The crystal packing exhibits weak inter­molecular C—H⋯S and C—H⋯N hydrogen bonds.

## Related literature

For applications of glycoluril derivatives, see: Wu *et al.* (2002 ▶). For a related structure, see: Wang & Xi (2009 ▶). For details of the synthesis, see: Ramos & Rosen (1981 ▶).
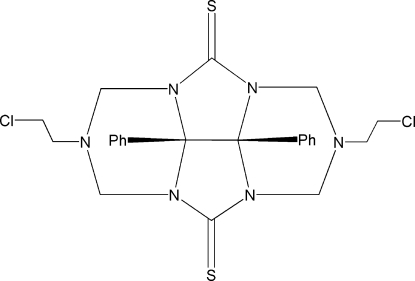


## Experimental

### 

#### Crystal data

C_24_H_26_Cl_2_N_6_S_2_
*M* *_r_* = 533.53
Monoclinic, 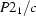

*a* = 8.7566 (2) Å
*b* = 14.0877 (3) Å
*c* = 20.8575 (5) Å
β = 99.525 (1)°
*V* = 2537.52 (10) Å^3^
*Z* = 4
Mo *K*α radiation
μ = 0.45 mm^−1^
*T* = 298 K
0.23 × 0.20 × 0.10 mm

#### Data collection

Bruker SMART 4K CCD area-detector diffractometer
Absorption correction: none
16129 measured reflections
4979 independent reflections
3375 reflections with *I* > 2σ(*I*)
*R*_int_ = 0.078

#### Refinement

*R*[*F* ^2^ > 2σ(*F* ^2^)] = 0.064
*wR*(*F* ^2^) = 0.164
*S* = 1.02
4979 reflections
307 parameters
H-atom parameters constrained
Δρ_max_ = 0.63 e Å^−3^
Δρ_min_ = −0.51 e Å^−3^

### 

Data collection: *SMART* (Bruker, 1997 ▶); cell refinement: *SAINT* (Bruker, 1999 ▶); data reduction: *SAINT*; program(s) used to solve structure: *SHELXS97* (Sheldrick, 2008 ▶); program(s) used to refine structure: *SHELXL97* (Sheldrick, 2008 ▶); molecular graphics: *SHELXTL* (Sheldrick, 2008 ▶); software used to prepare material for publication: *SHELXL97*.

## Supplementary Material

Crystal structure: contains datablocks I, global. DOI: 10.1107/S1600536809024118/cv2576sup1.cif

Structure factors: contains datablocks I. DOI: 10.1107/S1600536809024118/cv2576Isup2.hkl

Additional supplementary materials:  crystallographic information; 3D view; checkCIF report

## Figures and Tables

**Table 1:** Hydrogen-bond geometry (Å, °)

*D*—H⋯*A*	*D*—H	H⋯*A*	*D*⋯*A*	*D*—H⋯*A*
C24—H24*B*⋯N1^i^	0.97	2.55	3.485 (4)	162
C22—H22*B*⋯S1^ii^	0.97	2.80	3.607 (3)	141

Symmetry codes: (i) 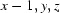; (ii) 
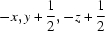
.

**Fig. 1.**
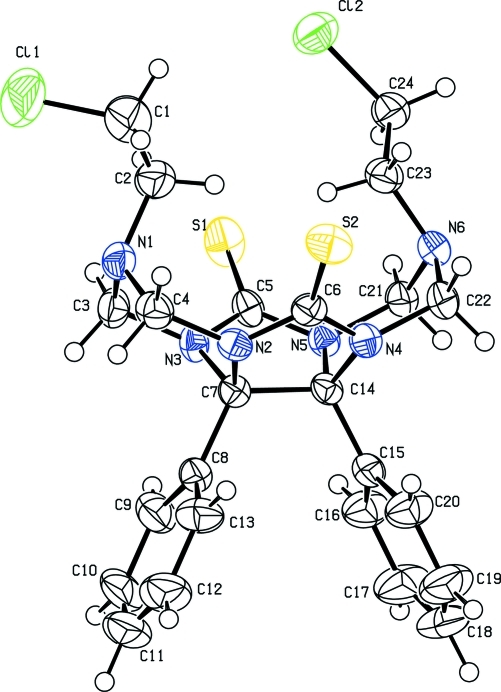
View of (I) showing the atom-labelling scheme. Displacement ellipsoids are drawn at the 50% probability level. H atoms are represented by spheres of arbitrary radius.

## supplementary crystallographic information

### Comment

Glycoluril derivatives have many areas of applications, such as explosives, slow-release fertilizers, cross-linkers, stabilizers of organic compounds against photodegradation, and as reagents in combinatorial chemistry (Wu *et al.*, 2002). Also, The rigid concave shape of glycoluril makes it a versatile building block to construct various supramolecular objects (Wang & Xi, 2009). We report here the structure of the title thioglycoluril derivative (Fig. 1), which is a potential receptor in supramolecular chemistry.

In the title compound all bond lengths and angles are normal and comparable with those observed in the related structure (Wang & Xi, 2009). The crystal packing is stabilized by intermolecular C–H···S and C—H···N hydrogen bonds (Table 1).

### Experimental

The title compound was synthesized according to the procedure reported by Ramos & Rosen (1981). Crystals appropriate for X-ray data collection were obtained by slow evaporation of the dichloromethane solution at 293 K.

### Refinement

All H atoms were initially located in a difference Fourier map and then included with constrained bond lengths and isotropic displacement parameters: C—H = 0.93 Å and Uĩso~(H) = 1.2 U~eq~(C) for aromatic H atoms, C—H = 0.97 Å and Uĩso~(H) = 1.2 U~eq~(C) for methylene H atoms, C—H = 0.96 Å and Uĩso~(H) = 1.5 U~eq~(C) for methyl H atoms.

### Crystal data

**Table:**

C_24_H_26_Cl_2_N_6_S_2_	*F*(000) = 1112
*M**_r_* = 533.53	*D*_x_ = 1.397 Mg m^−^^3^
Monoclinic, *P*2_1_/*c*	Mo *K*α radiation, λ = 0.71073 Å
Hall symbol: -P 2ybc	Cell parameters from 2851 reflections
*a* = 8.7566 (2) Å	θ = 2.5–21.7°
*b* = 14.0877 (3) Å	µ = 0.45 mm^−^^1^
*c* = 20.8575 (5) Å	*T* = 298 K
β = 99.525 (1)°	Block, colourless
*V* = 2537.52 (10) Å^3^	0.23 × 0.20 × 0.10 mm
*Z* = 4	

### Data collection

**Table:**

Bruker SMART 4K CCD area-detector diffractometer	3375 reflections with *I* > 2σ(*I*)
Radiation source: fine-focus sealed tube	*R*_int_ = 0.078
graphite	θ_max_ = 26.0°, θ_min_ = 1.8°
φ and ω scans	*h* = −10→10
16129 measured reflections	*k* = −17→17
4979 independent reflections	*l* = −18→25

### Refinement

**Table:**

Refinement on *F*^2^	Primary atom site location: structure-invariant direct methods
Least-squares matrix: full	Secondary atom site location: difference Fourier map
*R*[*F*^2^ > 2σ(*F*^2^)] = 0.064	Hydrogen site location: inferred from neighbouring sites
*wR*(*F*^2^) = 0.164	H-atom parameters constrained
*S* = 1.02	*w* = 1/[σ^2^(*F*_o_^2^) + (0.0798*P*)^2^] where *P* = (*F*_o_^2^ + 2*F*_c_^2^)/3
4979 reflections	(Δ/σ)_max_ < 0.001
307 parameters	Δρ_max_ = 0.63 e Å^−^^3^
0 restraints	Δρ_min_ = −0.51 e Å^−^^3^

### Special details

**Table:**

Geometry. All e.s.d.'s (except the e.s.d. in the dihedral angle between two l.s. planes) are estimated using the full covariance matrix. The cell e.s.d.'s are taken into account individually in the estimation of e.s.d.'s in distances, angles and torsion angles; correlations between e.s.d.'s in cell parameters are only used when they are defined by crystal symmetry. An approximate (isotropic) treatment of cell e.s.d.'s is used for estimating e.s.d.'s involving l.s. planes.
Refinement. Refinement of *F*^2^ against ALL reflections. The weighted *R*-factor *wR* and goodness of fit *S* are based on *F*^2^, conventional *R*-factors *R* are based on *F*, with *F* set to zero for negative *F*^2^. The threshold expression of *F*^2^ > σ(*F*^2^) is used only for calculating *R*-factors(gt) *etc*. and is not relevant to the choice of reflections for refinement. *R*-factors based on *F*^2^ are statistically about twice as large as those based on *F*, and *R*- factors based on ALL data will be even larger.

### Fractional atomic coordinates and isotropic or equivalent isotropic displacement parameters (Å^2^)

**Table:**

	*x*	*y*	*z*	*U*_iso_*/*U*_eq_	
C1	0.5265 (5)	0.0996 (4)	0.4154 (2)	0.0757 (13)	
H1A	0.4497	0.0898	0.4434	0.091*	
H1B	0.5458	0.1673	0.4136	0.091*	
C2	0.4639 (4)	0.0657 (3)	0.34973 (18)	0.0501 (9)	
H2A	0.4466	−0.0022	0.3516	0.060*	
H2B	0.3642	0.0956	0.3356	0.060*	
C3	0.5429 (3)	0.0178 (2)	0.24750 (17)	0.0394 (8)	
H3A	0.5366	−0.0461	0.2641	0.047*	
H3B	0.6324	0.0210	0.2257	0.047*	
C4	0.5586 (4)	0.1811 (2)	0.27789 (16)	0.0393 (8)	
H4A	0.6483	0.1925	0.2572	0.047*	
H4B	0.5643	0.2242	0.3145	0.047*	
C5	0.2613 (3)	−0.0038 (2)	0.19965 (15)	0.0319 (7)	
C6	0.2848 (3)	0.2397 (2)	0.24636 (15)	0.0314 (7)	
C7	0.3878 (3)	0.13561 (19)	0.17632 (14)	0.0281 (7)	
C8	0.4933 (3)	0.1550 (2)	0.12701 (15)	0.0342 (7)	
C9	0.5363 (4)	0.0818 (3)	0.08934 (17)	0.0493 (9)	
H9	0.5021	0.0203	0.0947	0.059*	
C10	0.6305 (5)	0.1006 (4)	0.0436 (2)	0.0679 (13)	
H10	0.6592	0.0516	0.0182	0.081*	
C11	0.6819 (5)	0.1918 (4)	0.0354 (2)	0.0725 (14)	
H11	0.7445	0.2041	0.0044	0.087*	
C12	0.6409 (4)	0.2640 (3)	0.0727 (2)	0.0670 (12)	
H12	0.6763	0.3253	0.0676	0.080*	
C13	0.5465 (4)	0.2455 (3)	0.11833 (18)	0.0494 (9)	
H13	0.5184	0.2949	0.1436	0.059*	
C14	0.2095 (3)	0.14683 (19)	0.15257 (14)	0.0278 (7)	
C15	0.1585 (3)	0.1754 (2)	0.08262 (15)	0.0336 (7)	
C16	0.1323 (4)	0.1075 (3)	0.03430 (17)	0.0496 (9)	
H16	0.1440	0.0436	0.0450	0.060*	
C17	0.0888 (5)	0.1341 (3)	−0.02984 (19)	0.0687 (12)	
H17	0.0719	0.0879	−0.0621	0.082*	
C18	0.0703 (5)	0.2282 (4)	−0.0463 (2)	0.0752 (14)	
H18	0.0392	0.2458	−0.0894	0.090*	
C19	0.0978 (5)	0.2958 (3)	0.0012 (2)	0.0714 (13)	
H19	0.0870	0.3597	−0.0098	0.086*	
C20	0.1416 (4)	0.2694 (3)	0.06534 (18)	0.0522 (10)	
H20	0.1599	0.3159	0.0973	0.063*	
C21	−0.0141 (3)	0.0516 (2)	0.17475 (16)	0.0345 (7)	
H21A	−0.0784	0.0657	0.1334	0.041*	
H21B	−0.0404	−0.0117	0.1876	0.041*	
C22	0.0005 (3)	0.2145 (2)	0.20563 (17)	0.0384 (8)	
H22A	−0.0628	0.2341	0.1652	0.046*	
H22B	−0.0163	0.2593	0.2391	0.046*	
C23	0.0225 (4)	0.0944 (2)	0.28981 (15)	0.0383 (8)	
H23A	0.0157	0.1485	0.3180	0.046*	
H23B	0.1313	0.0806	0.2906	0.046*	
C24	−0.0531 (4)	0.0101 (2)	0.31604 (17)	0.0429 (8)	
H24A	−0.0589	−0.0425	0.2857	0.052*	
H24B	−0.1573	0.0262	0.3223	0.052*	
Cl1	0.69817 (17)	0.04249 (16)	0.44949 (7)	0.1337 (8)	
Cl2	0.06244 (11)	−0.02216 (7)	0.39222 (5)	0.0585 (3)	
N1	0.5631 (3)	0.08436 (18)	0.30145 (13)	0.0382 (7)	
N2	0.4172 (3)	0.20160 (16)	0.23108 (12)	0.0304 (6)	
N3	0.4014 (3)	0.03855 (16)	0.20014 (13)	0.0301 (6)	
N4	0.1644 (3)	0.21709 (16)	0.19809 (12)	0.0303 (6)	
N5	0.1496 (3)	0.05305 (16)	0.16642 (12)	0.0289 (6)	
N6	−0.0485 (3)	0.11945 (18)	0.22321 (13)	0.0356 (6)	
S1	0.23332 (10)	−0.10921 (6)	0.23090 (5)	0.0516 (3)	
S2	0.27222 (11)	0.30702 (6)	0.31074 (4)	0.0461 (3)	

### Atomic displacement parameters (Å^2^)

**Table:**

	*U*^11^	*U*^22^	*U*^33^	*U*^12^	*U*^13^	*U*^23^
C1	0.065 (3)	0.104 (4)	0.059 (3)	0.015 (3)	0.015 (2)	0.001 (3)
C2	0.041 (2)	0.064 (2)	0.045 (2)	−0.0041 (17)	0.0059 (17)	0.0122 (19)
C3	0.0314 (17)	0.0371 (18)	0.049 (2)	0.0063 (14)	0.0052 (16)	0.0086 (16)
C4	0.0339 (18)	0.0444 (19)	0.040 (2)	−0.0068 (14)	0.0066 (15)	−0.0017 (16)
C5	0.0287 (16)	0.0280 (16)	0.040 (2)	0.0012 (12)	0.0087 (14)	−0.0048 (14)
C6	0.0374 (17)	0.0230 (15)	0.0350 (19)	0.0003 (12)	0.0097 (15)	0.0045 (13)
C7	0.0300 (16)	0.0241 (15)	0.0307 (17)	0.0008 (12)	0.0068 (13)	−0.0010 (13)
C8	0.0279 (16)	0.0418 (19)	0.0333 (18)	−0.0004 (13)	0.0061 (14)	0.0005 (15)
C9	0.042 (2)	0.061 (2)	0.047 (2)	0.0026 (17)	0.0154 (18)	−0.0076 (19)
C10	0.050 (2)	0.112 (4)	0.046 (3)	0.015 (2)	0.023 (2)	−0.014 (2)
C11	0.047 (2)	0.124 (4)	0.051 (3)	−0.011 (3)	0.021 (2)	0.016 (3)
C12	0.057 (3)	0.088 (3)	0.058 (3)	−0.022 (2)	0.015 (2)	0.019 (3)
C13	0.057 (2)	0.051 (2)	0.043 (2)	−0.0113 (17)	0.0161 (18)	0.0069 (17)
C14	0.0305 (16)	0.0233 (15)	0.0313 (17)	0.0030 (12)	0.0105 (13)	−0.0017 (13)
C15	0.0329 (17)	0.0358 (17)	0.0340 (19)	0.0058 (13)	0.0113 (14)	−0.0005 (14)
C16	0.070 (3)	0.044 (2)	0.036 (2)	0.0072 (18)	0.0130 (19)	−0.0031 (17)
C17	0.102 (4)	0.073 (3)	0.031 (2)	0.007 (3)	0.011 (2)	−0.009 (2)
C18	0.093 (4)	0.102 (4)	0.032 (2)	0.020 (3)	0.016 (2)	0.022 (3)
C19	0.103 (4)	0.063 (3)	0.051 (3)	0.023 (2)	0.019 (3)	0.024 (2)
C20	0.073 (3)	0.042 (2)	0.043 (2)	0.0065 (18)	0.012 (2)	0.0064 (18)
C21	0.0276 (16)	0.0409 (18)	0.0349 (19)	−0.0010 (13)	0.0048 (14)	0.0002 (15)
C22	0.0340 (17)	0.0412 (19)	0.041 (2)	0.0118 (14)	0.0105 (15)	−0.0007 (16)
C23	0.0374 (18)	0.0450 (19)	0.034 (2)	−0.0018 (15)	0.0086 (15)	−0.0027 (15)
C24	0.0354 (18)	0.053 (2)	0.041 (2)	−0.0052 (15)	0.0065 (16)	0.0053 (17)
Cl1	0.0874 (10)	0.253 (2)	0.0537 (8)	0.0758 (12)	−0.0083 (7)	0.0002 (10)
Cl2	0.0556 (6)	0.0663 (6)	0.0516 (6)	−0.0015 (5)	0.0029 (5)	0.0170 (5)
N1	0.0297 (14)	0.0429 (16)	0.0413 (17)	0.0002 (12)	0.0033 (12)	0.0062 (13)
N2	0.0316 (14)	0.0296 (13)	0.0307 (15)	−0.0033 (11)	0.0076 (11)	−0.0015 (11)
N3	0.0241 (13)	0.0236 (13)	0.0426 (16)	0.0012 (10)	0.0058 (11)	0.0019 (11)
N4	0.0342 (14)	0.0255 (13)	0.0319 (15)	0.0052 (10)	0.0077 (12)	−0.0022 (11)
N5	0.0270 (13)	0.0264 (13)	0.0340 (15)	−0.0008 (10)	0.0074 (11)	0.0005 (11)
N6	0.0320 (14)	0.0409 (16)	0.0356 (16)	0.0058 (11)	0.0105 (12)	0.0000 (12)
S1	0.0454 (5)	0.0260 (5)	0.0856 (8)	−0.0009 (4)	0.0169 (5)	0.0123 (5)
S2	0.0600 (6)	0.0389 (5)	0.0420 (6)	−0.0011 (4)	0.0163 (5)	−0.0135 (4)

### Geometric parameters (Å, °)

**Table:**

C1—C2	1.469 (6)	C12—C13	1.384 (5)
C1—Cl1	1.749 (4)	C12—H12	0.9300
C1—H1A	0.9700	C13—H13	0.9300
C1—H1B	0.9700	C14—N5	1.468 (3)
C2—N1	1.459 (4)	C14—N4	1.470 (3)
C2—H2A	0.9700	C14—C15	1.508 (4)
C2—H2B	0.9700	C15—C20	1.374 (4)
C3—N1	1.453 (4)	C15—C16	1.381 (4)
C3—N3	1.480 (4)	C16—C17	1.381 (5)
C3—H3A	0.9700	C16—H16	0.9300
C3—H3B	0.9700	C17—C18	1.372 (6)
C4—N1	1.447 (4)	C17—H17	0.9300
C4—N2	1.473 (4)	C18—C19	1.366 (6)
C4—H4A	0.9700	C18—H18	0.9300
C4—H4B	0.9700	C19—C20	1.381 (5)
C5—N5	1.362 (4)	C19—H19	0.9300
C5—N3	1.363 (4)	C20—H20	0.9300
C5—S1	1.656 (3)	C21—N6	1.458 (4)
C6—N2	1.362 (4)	C21—N5	1.472 (4)
C6—N4	1.369 (4)	C21—H21A	0.9700
C6—S2	1.662 (3)	C21—H21B	0.9700
C7—N3	1.453 (3)	C22—N4	1.470 (4)
C7—N2	1.462 (4)	C22—N6	1.471 (4)
C7—C8	1.517 (4)	C22—H22A	0.9700
C7—C14	1.565 (4)	C22—H22B	0.9700
C8—C13	1.379 (4)	C23—N6	1.468 (4)
C8—C9	1.385 (4)	C23—C24	1.506 (4)
C9—C10	1.386 (5)	C23—H23A	0.9700
C9—H9	0.9300	C23—H23B	0.9700
C10—C11	1.381 (6)	C24—Cl2	1.795 (3)
C10—H10	0.9300	C24—H24A	0.9700
C11—C12	1.364 (6)	C24—H24B	0.9700
C11—H11	0.9300		
			
C2—C1—Cl1	113.1 (3)	C20—C15—C16	118.6 (3)
C2—C1—H1A	109.0	C20—C15—C14	120.8 (3)
Cl1—C1—H1A	109.0	C16—C15—C14	120.5 (3)
C2—C1—H1B	109.0	C17—C16—C15	120.4 (3)
Cl1—C1—H1B	109.0	C17—C16—H16	119.8
H1A—C1—H1B	107.8	C15—C16—H16	119.8
N1—C2—C1	114.3 (3)	C18—C17—C16	120.4 (4)
N1—C2—H2A	108.7	C18—C17—H17	119.8
C1—C2—H2A	108.7	C16—C17—H17	119.8
N1—C2—H2B	108.7	C19—C18—C17	119.6 (4)
C1—C2—H2B	108.7	C19—C18—H18	120.2
H2A—C2—H2B	107.6	C17—C18—H18	120.2
N1—C3—N3	111.8 (2)	C18—C19—C20	120.1 (4)
N1—C3—H3A	109.3	C18—C19—H19	120.0
N3—C3—H3A	109.3	C20—C19—H19	120.0
N1—C3—H3B	109.3	C15—C20—C19	121.0 (4)
N3—C3—H3B	109.3	C15—C20—H20	119.5
H3A—C3—H3B	107.9	C19—C20—H20	119.5
N1—C4—N2	112.5 (2)	N6—C21—N5	112.9 (2)
N1—C4—H4A	109.1	N6—C21—H21A	109.0
N2—C4—H4A	109.1	N5—C21—H21A	109.0
N1—C4—H4B	109.1	N6—C21—H21B	109.0
N2—C4—H4B	109.1	N5—C21—H21B	109.0
H4A—C4—H4B	107.8	H21A—C21—H21B	107.8
N5—C5—N3	108.4 (2)	N4—C22—N6	112.3 (2)
N5—C5—S1	126.0 (2)	N4—C22—H22A	109.1
N3—C5—S1	125.5 (2)	N6—C22—H22A	109.1
N2—C6—N4	108.5 (3)	N4—C22—H22B	109.1
N2—C6—S2	125.9 (2)	N6—C22—H22B	109.1
N4—C6—S2	125.5 (2)	H22A—C22—H22B	107.9
N3—C7—N2	109.7 (2)	N6—C23—C24	113.0 (3)
N3—C7—C8	112.1 (2)	N6—C23—H23A	109.0
N2—C7—C8	111.5 (2)	C24—C23—H23A	109.0
N3—C7—C14	103.0 (2)	N6—C23—H23B	109.0
N2—C7—C14	102.7 (2)	C24—C23—H23B	109.0
C8—C7—C14	117.1 (2)	H23A—C23—H23B	107.8
C13—C8—C9	118.9 (3)	C23—C24—Cl2	107.7 (2)
C13—C8—C7	120.8 (3)	C23—C24—H24A	110.2
C9—C8—C7	120.3 (3)	Cl2—C24—H24A	110.2
C10—C9—C8	119.8 (4)	C23—C24—H24B	110.2
C10—C9—H9	120.1	Cl2—C24—H24B	110.2
C8—C9—H9	120.1	H24A—C24—H24B	108.5
C11—C10—C9	120.4 (4)	C4—N1—C3	110.6 (3)
C11—C10—H10	119.8	C4—N1—C2	114.7 (3)
C9—C10—H10	119.8	C3—N1—C2	114.1 (3)
C12—C11—C10	120.0 (4)	C6—N2—C7	112.7 (2)
C12—C11—H11	120.0	C6—N2—C4	125.2 (3)
C10—C11—H11	120.0	C7—N2—C4	114.4 (2)
C11—C12—C13	119.7 (4)	C5—N3—C7	112.7 (2)
C11—C12—H12	120.1	C5—N3—C3	124.9 (3)
C13—C12—H12	120.1	C7—N3—C3	115.2 (2)
C8—C13—C12	121.2 (4)	C6—N4—C14	112.0 (2)
C8—C13—H13	119.4	C6—N4—C22	125.0 (3)
C12—C13—H13	119.4	C14—N4—C22	115.1 (2)
N5—C14—N4	109.6 (2)	C5—N5—C14	112.3 (2)
N5—C14—C15	111.8 (2)	C5—N5—C21	124.3 (2)
N4—C14—C15	112.1 (2)	C14—N5—C21	115.0 (2)
N5—C14—C7	102.5 (2)	C21—N6—C23	113.2 (2)
N4—C14—C7	102.9 (2)	C21—N6—C22	108.8 (2)
C15—C14—C7	117.0 (2)	C23—N6—C22	111.1 (3)
			
Cl1—C1—C2—N1	−63.9 (4)	C8—C7—N2—C6	132.9 (3)
N3—C7—C8—C13	−153.4 (3)	C14—C7—N2—C6	6.7 (3)
N2—C7—C8—C13	−29.9 (4)	N3—C7—N2—C4	48.9 (3)
C14—C7—C8—C13	88.0 (4)	C8—C7—N2—C4	−75.9 (3)
N3—C7—C8—C9	27.4 (4)	C14—C7—N2—C4	157.9 (2)
N2—C7—C8—C9	150.9 (3)	N1—C4—N2—C6	93.6 (3)
C14—C7—C8—C9	−91.2 (3)	N1—C4—N2—C7	−53.5 (3)
C13—C8—C9—C10	−0.4 (5)	N5—C5—N3—C7	10.2 (3)
C7—C8—C9—C10	178.8 (3)	S1—C5—N3—C7	−172.8 (2)
C8—C9—C10—C11	0.1 (6)	N5—C5—N3—C3	158.9 (3)
C9—C10—C11—C12	0.4 (7)	S1—C5—N3—C3	−24.0 (4)
C10—C11—C12—C13	−0.6 (7)	N2—C7—N3—C5	103.0 (3)
C9—C8—C13—C12	0.2 (5)	C8—C7—N3—C5	−132.5 (3)
C7—C8—C13—C12	−179.0 (3)	C14—C7—N3—C5	−5.8 (3)
C11—C12—C13—C8	0.3 (6)	N2—C7—N3—C3	−48.9 (3)
N3—C7—C14—N5	−0.4 (3)	C8—C7—N3—C3	75.5 (3)
N2—C7—C14—N5	−114.4 (2)	C14—C7—N3—C3	−157.8 (2)
C8—C7—C14—N5	123.1 (3)	N1—C3—N3—C5	−95.3 (3)
N3—C7—C14—N4	113.5 (2)	N1—C3—N3—C7	52.8 (3)
N2—C7—C14—N4	−0.6 (3)	N2—C6—N4—C14	10.1 (3)
C8—C7—C14—N4	−123.1 (3)	S2—C6—N4—C14	−172.8 (2)
N3—C7—C14—C15	−123.1 (3)	N2—C6—N4—C22	157.3 (2)
N2—C7—C14—C15	122.9 (3)	S2—C6—N4—C22	−25.5 (4)
C8—C7—C14—C15	0.3 (4)	N5—C14—N4—C6	102.9 (3)
N5—C14—C15—C20	151.7 (3)	C15—C14—N4—C6	−132.2 (3)
N4—C14—C15—C20	28.1 (4)	C7—C14—N4—C6	−5.6 (3)
C7—C14—C15—C20	−90.5 (3)	N5—C14—N4—C22	−47.8 (3)
N5—C14—C15—C16	−30.2 (4)	C15—C14—N4—C22	77.1 (3)
N4—C14—C15—C16	−153.9 (3)	C7—C14—N4—C22	−156.4 (2)
C7—C14—C15—C16	87.5 (4)	N6—C22—N4—C6	−92.4 (3)
C20—C15—C16—C17	−0.5 (5)	N6—C22—N4—C14	54.0 (3)
C14—C15—C16—C17	−178.6 (3)	N3—C5—N5—C14	−10.4 (3)
C15—C16—C17—C18	−0.3 (6)	S1—C5—N5—C14	172.6 (2)
C16—C17—C18—C19	1.1 (7)	N3—C5—N5—C21	−156.7 (3)
C17—C18—C19—C20	−1.0 (7)	S1—C5—N5—C21	26.3 (4)
C16—C15—C20—C19	0.7 (5)	N4—C14—N5—C5	−102.4 (3)
C14—C15—C20—C19	178.7 (3)	C15—C14—N5—C5	132.6 (3)
C18—C19—C20—C15	0.1 (7)	C7—C14—N5—C5	6.5 (3)
N6—C23—C24—Cl2	171.8 (2)	N4—C14—N5—C21	47.2 (3)
N2—C4—N1—C3	54.4 (3)	C15—C14—N5—C21	−77.8 (3)
N2—C4—N1—C2	−76.3 (3)	C7—C14—N5—C21	156.1 (2)
N3—C3—N1—C4	−53.6 (3)	N6—C21—N5—C5	92.0 (3)
N3—C3—N1—C2	77.5 (3)	N6—C21—N5—C14	−53.5 (3)
C1—C2—N1—C4	−77.7 (4)	N5—C21—N6—C23	−68.8 (3)
C1—C2—N1—C3	153.3 (3)	N5—C21—N6—C22	55.2 (3)
N4—C6—N2—C7	−10.6 (3)	C24—C23—N6—C21	−71.7 (3)
S2—C6—N2—C7	172.3 (2)	C24—C23—N6—C22	165.6 (2)
N4—C6—N2—C4	−158.2 (3)	N4—C22—N6—C21	−55.4 (3)
S2—C6—N2—C4	24.7 (4)	N4—C22—N6—C23	69.9 (3)
N3—C7—N2—C6	−102.3 (3)		

### Hydrogen-bond geometry (Å, °)

**Table:**

*D*—H···*A*	*D*—H	H···*A*	*D*···*A*	*D*—H···*A*
C24—H24B···N1^i^	0.97	2.55	3.485 (4)	162
C22—H22B···S1^ii^	0.97	2.80	3.607 (3)	141

Symmetry codes: (i) *x*−1, *y*, *z*; (ii) −*x*, *y*+1/2, −*z*+1/2.
